# Intermittent Propofol Exposure Induces Neurodevelopmental Alterations in Human Brain Organoids

**DOI:** 10.1007/s10571-026-01673-2

**Published:** 2026-01-26

**Authors:** Sudena Wang, Chloe Hall, Yong Wang, Leonie Link, Yi Zhang, Alexander Schlägel, Cora Wunder, Christopher Patzke, Matthias Klein, Thomas Mittmann, Michael K. E. Schäfer

**Affiliations:** 1 Department of Anesthesiology, University Medical Center, Johannes Gutenberg-University Mainz, Mainz, Germany; 2 Institute for Physiology, University Medical Centre of the Johannes Gutenberg University Mainz, Mainz, Germany; 3 Department of Plastic Surgery, Renmin Hospital of Wuhan University, Wuhan, 430060 Hubei China; 4 Institute of Legal Medicine, Johannes-Gutenberg University Mainz, 55131 Mainz, Germany; 5 Department of Biological Science, University of Notre Dame, Notre Dame, IN USA; 6 Institute for Immunology, University Medical Center, Johannes Gutenberg-University Mainz, Langenbeckstr. 1, 55131 Mainz, Germany; 7 Focus Program Translational Neurosciences (FTN) of the Johannes Gutenberg-University Mainz, Langenbeckstr. 1, 55131 Mainz, Germany; 8 Research Center for Immunotherapy (FZI), Johannes Gutenberg-University Mainz, Langenbeckstr. 1, 55131 Mainz, Germany

**Keywords:** Human brain organoids, Human cerebral organoids, Anesthetics, Propofol, Neurodevelopment

## Abstract

The administration of anaesthesia during pregnancy may have implications for fetal brain development. This study used H1 embryonic stem cell-derived human brain organoids (HBOs) to investigate effects of intermittent propofol exposure (IPE). HBOs were subjected to early IPE from 47 to 50 days in vitro (div), or late IPE from 77 to 80 div, using a clinically supra-anaesthetic concentration of 50 µM propofol. This was followed by cultivation without propofol for an additional 10 div, and HBOs were subsequently analysed at 60 or 90 div. Determination of HBO growth and lactate release did not provide evidence of neurotoxicity. Multi-electrode array recordings indicated an increased neuronal activity at 60 div following early IPE, an effect not observed at 90 div following late IPE. RNA-sequencing revealed that IPE up-regulated genes associated with neurodevelopment and synapse functions at 60 div, which overlapped with naturally up-regulated genes during HBO development from 60 to 90 div. These findings indicate that early IPE accelerates brain maturation in HBOs, suggesting possible deviations from the normal developmental trajectory in the fetal brain.

**Graphical Abstract:**

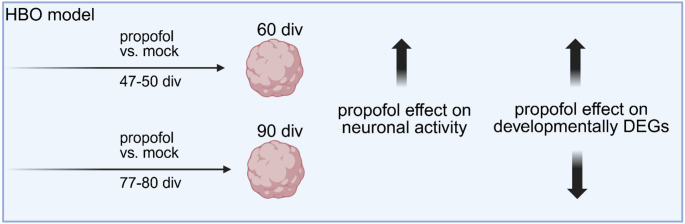

**Supplementary Information:**

The online version contains supplementary material available at 10.1007/s10571-026-01673-2.

## Introduction

Brain development is a multifaceted process involving neurogenesis, neuronal migration and differentiation, synaptogenesis, and synaptic plasticity, all essential to establish and maintain neural networks (Budday et al. 2015). The developing brain may encounter environmental challenges, including anaesthetics, which impact neural networks and force adaptive changes (Sen 2016). The challenges arising from the vulnerability of the developing brain to anaesthetics are particularly important during pregnancy and in premature infants and neonates (Paulus 2005; Ruggiero et al. 2019). Millions of infants and children worldwide are exposed to anaesthetics each year, and between 0.75% and 2% of pregnant women receive anaesthetics because of non-obstetric surgery (Reitman and Flood 2011; Zhou et al. 2023).

Propofol is a widely used intravenous general anaesthetic for inducing and maintaining anaesthesia, and for sedation in intensive care, including pregnant women (Bosnjak et al. 2016; Malhotra et al. 2017; Zhang and Li 2023). A fast placenta transfer with venous blood concentration mother-to-fetus ratio ranging from 0.7 to 0.8 has been reported (Dailland et al. 1989; Sahinovic et al. 2018), suggesting a potential impact on brain development. Although propofol or other anaesthetics have not been definitively proven harmful to the human fetus, evidence indicates that exposure to anaesthetics may adversely affect neurodevelopment and cognitive outcome (Ing and Vutskits 2023; Reighard et al. 2022) and neurotoxic effects have been reported in humans and experimental in vivo and in vitro approaches across a wide range of concentrations and context-dependent conditions (Bosnjak et al. 2016; Zhang and Li 2023). Ethical constraints preclude randomized trials on pregnant patients, and no animal model adequately replicates human gestation (Reitman and Flood 2011). Nevertheless, the pathological effects of prenatal and neonatal anaesthesia have been investigated in rodent models. Exposure to anaesthesia, and in particularpropofol, at an early age can lead to behavioral changes, and cognitive dysfunction (Aksenov et al. 2020; Chen et al. 2016; Sen and Sen 2016). Experimental studies demonstrated compromised neurogenesis, neuronal migration, as well as neurotoxic and neuroapoptotic effects in different brain structures (Gascoigne et al. 2021; Gluncic et al. 2019; Hirotsu et al. 2019; Yan et al. 2017). Moreover, the effects of propofol have been examined in a series of studies in rats at postnatal day 7, revealing cell death and the activation of cell death-associated-pathways. This was evidenced by the up-regulation of calpain, caspases, FasL, as well as the inhibition of anti-apoptotic Bcl2 mRNA and protein, respectively (Milanovic et al. 2010, 2016). Moreover, the administration of propofol resulted in a reduction of synaptic protein expression, as well as alterations in synaptic structure and connectivity. At the same time, the upregulation of the axonal growth and plasticity marker GAP-43 and the dendritic microtubule-stabilizing protein MAP-2 indicated the activation of compensatory mechanisms in response to propofol-induced neurotoxicity (Milanovic et al. 2017). Certain processes appeared to be restricted to specific brain regions, such as increased synaptogenesis in the frontal cortex and synaptic elimination in the thalamus. These findings suggested both immediate and lasting effects of propofol administration during critical phases of brain development (Pešić et al. 2015). Similar results have been reported in rats whose offspring were investigated after maternal propofol administration during pregnancy, including reduced hippocampal neuronal cell density and synaptophysin expression (Xiong et al. 2014). Early postnatal administration of propofol and maternal exposure to propofol was also shown to induce cell death in rhesus macaque brain (Creeley et al. 2013) and have been associated with lasting behavioral alterations in rats (Chen et al. 2016; Li et al. 2014; Milanovic et al. 2016, 2017; Pešić et al. 2015; Wan et al. 2021; Xiong et al. 2014; Zhong et al. 2016).

Human brain organoids (HBOs) derived from human embryonic stem cell (hESC) or induced pluripotent stem cell (iPSC) lines are considered suitable models to investigate various aspects of human fetal brain development, including the maturation of neural networks (Benito-Kwiecinski and Lancaster 2020; Qian et al. 2019; Zafeiriou et al. 2020). The term HBO is a broad term for any three-dimensional neural organoid culture, including both directed, brain region-specific organoids and spontaneous, self-organizing three-dimensional neural organoid cultures, which are often interchangeably called cerebral organoids (Qian et al. 2019).

Although anaesthesia may adversely affect fetal brain development, only few studies have investigated anaesthetics in ESC- or iPSC-derived HBOs. The findings indicated premature neuronal differentiation following short-term exposure to sevoflurane (Lee et al. 2022; Shang et al. 2022) as well as neurotoxic effects of chronic ketamine exposure (Du et al. 2023) and high dosage propofol exposure (Saglam-Metiner et al. 2024). However, propofol was also shown to elicit functional electrophysiological responses in HBOs (Logan et al. 2020). Overall, the knowledge on the effects of anaesthetics on HBOs is scarce. In particular, developmental stage-dependent effects of propofol, one of the most common anaesthetics used over prolonged periods, have not yet been investigated in the HBO model of fetal brain development. This model may offer new insights that may be relevant for evaluating the risk of potential adverse effects in clinical situations involving prolonged anesthesia during pregnancy. To experimentally address this issue, we investigated effects of prolonged intermittent propofol exposure (IPE) over two different time points during HBO development, two- and three-months-old HBO cultures, considered to mimic in vivo early- to mid-fetal brain development (Zhao and Haddad 2024). Combining electrophysiological recordings via multi-electrode arrays (MEAs), bulk RNA-sequencing, the subsequent identification of differentially expressed genes (DEGs) and pathways, and gene set enrichment analysis (GSEA), we present evidence that IPE induces developmental stage-specific alterations in neuronal activity and synapse-associated gene expression in HBOs.

## Methods

### Study Design

**Fig. 1:**
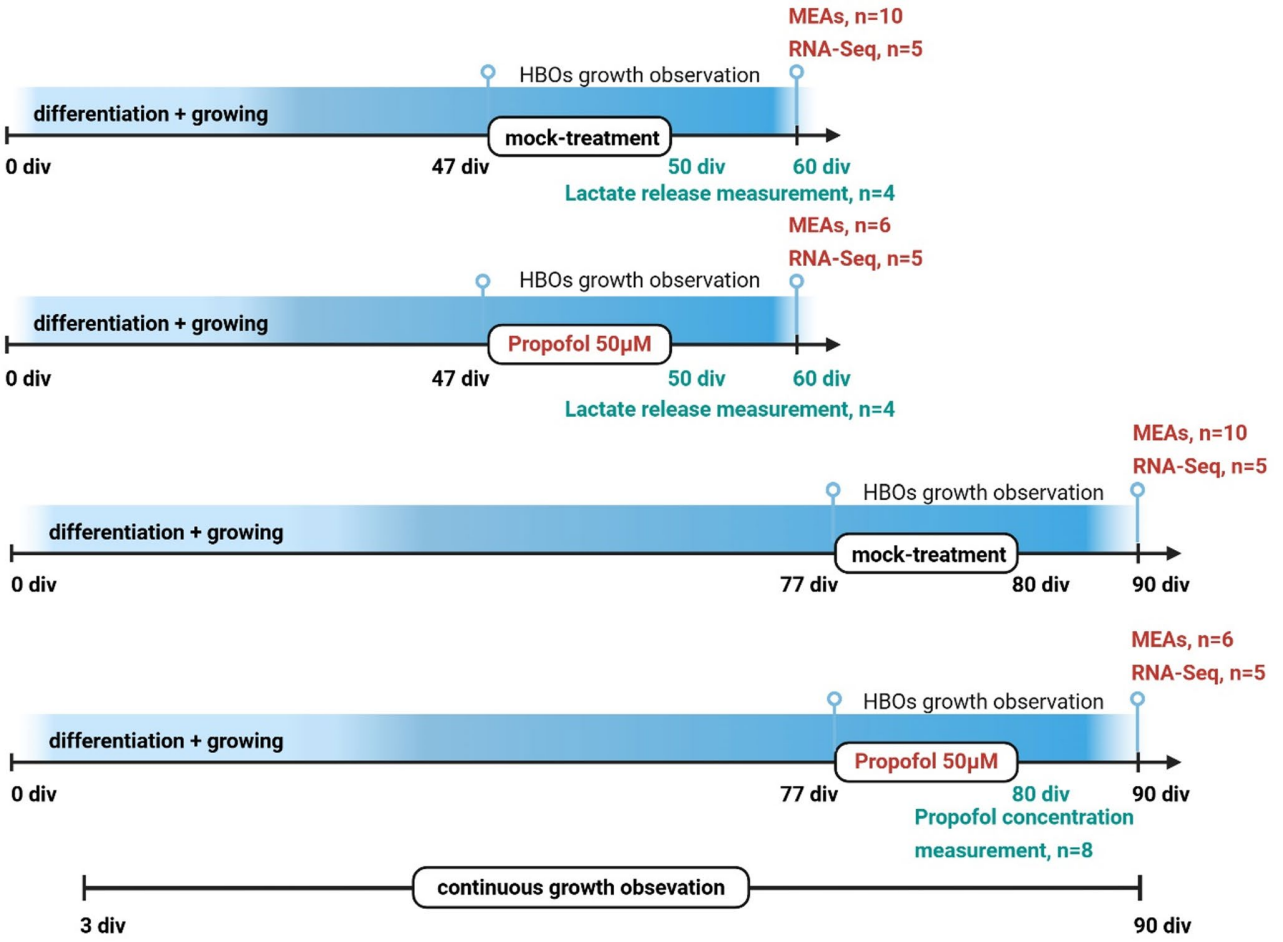
Study design. A total of 68 HBOs was investigated in four experimental groups at two developmental stages, 60 div and 90 div, including mock-treated groups as control and propofol-treated groups in which HBOs were intermittently exposed to 50 µM of propofol for 3 div (from 47–50 div, or from 77–80 div). HBOs were analysed using MEAs (*n* = 32, two batches), processed for RNA-Seq (*n* = 20, two batches), propofol concentration measurements (*n* = 8, two batches), and lactate concentrations were determined in HBO medium (*n* = 8, one batch)

A total of 68 HBOs were investigated in four experimental groups at two developmental stages (Fig. 1), 60 div and 90 div, presumed to exhibit neuronal activity (Fair et al. 2020; Trujillo et al. 2019), including mock-treated groups as control and propofol-treated groups in which HBOs were intermittently exposed to 50 µM of propofol for 3 div (from 47 to 50 div, or from 77 to 80 div). This concentration was selected based on findings from clinical human brain tissue concentrations (Van Hese et al. 2020), and studies on human iPSC-derived neural progenitor cells (NPCs), which indicated no immediate or delayed toxicity after a 6-hour exposure to 50 µM propofol (Long et al. 2017). We examined the effects of IPE to HBOs at different developmental stages, determined propofol concentrations in HBOs (*n* = 8, technical replicates from one batch), and investigated HBO size and growth (*n* = 10, technical replicates from two batches), release of the energy metabolite lactate (*n* = 8, technical replicates from one batch), neuronal activity via multi-electrode arrays (MEAs) (*n* = 32, technical replicates from two batches), as well as identifying differentially expressed genes (DEGs) and associated pathways and gene set enrichment analysis (GSEA) following bulk RNA-sequencing (*n* = 20, technical replicates from two batches.

### Generation of HBOs

Brain organoids were generated from the male human embryonic stem cell line H1 (H1-hESC, WA01, WiCell Research Institute, NIH registration/approval number: 0043/NIHhESC-10–0043) according to a previous protocol with slight modifications (Sutcliffe and Lancaster 2019). Briefly, H1-hESCs were cultured at passage 45 on solidified medium composed of Matrigel/MEM and mTeSR1 (StemCell) until they reached approximately 70–80% confluency in the 6-well plate. On day 0, the cells were detached using Accutase (Sigma-Aldrich) and seeded into a 96-well Ultra Low Attachment plate (ULA, Corning) at a density of 9000 cells per well in 150 µL of mTeSR1, supplemented with 4 ng/ml bFGF (Peprotech) and 1 µM Thiazovivin (Sigma-Aldrich). On day 3 in vitro (3 div), 75 µl of the medium was replaced with 150 µL of fresh mTeSR1 without any of bFGF or Thiazovivin. On 6 div, embryoid bodies had formed and were transferred to a 24-well ULA plate containing 500 µL of neural induction medium (supplementary material, Table 1). Based on their morphology and size, on 12 div, the organoids were coated with Matrigel (Corning) and further cultured in 60 mm petri dishes (Greiner) with differentiation medium lacking retinoic acid (supplementary material, Table 1) on an orbital shaker (Edmund Bühler) until the end of the experiment on either 60 div or 90 div. At 16 div, the Matrigel coating was removed gently, and the organoids were cultured in differentiation medium containing retinoic acid (supplementary material, Table 1). All the incubation phases were conducted at 37 °C with 5% CO2.

### Intermittent Exposure of HBOs to Propofol

Propofol (LGC, Wesel Germany) was diluted into dimethylsulfoxide (DMSO) to obtain a 10 mM stock solution. A concentration of 50 µM propofol from this stock solution was added into the culture medium at 47 div or 77 div and maintained for a duration of 72 h (3 div), followed by replacement with fresh medium w/o propofol for 10 div. In the control condition, an equivalent volume of the DMSO was added (final concentration: 0.5% DMSO), serving as a mock-treatment (Fig. 1).

### Analysis of Propofol Concentration in HBOs

A propofol stock solution (1 mg/ml, LGC, Wesel Germany) with a final concentration of 2 ng/µl was prepared. The further calibrators were diluted from this solution. Calibration comprised 7 calibrators with concentrations of 1, 2, 3, 4, 5, 10 and 20 ng/mg. For preparation of each calibrator, 10 µl of the respective standard solution was transferred to 10 mg of brain (human brain blank matrix). Subsequently, 10 µl of the internal standard (ISTD) Propofol-D_17_ (LGC, Wesel Germany) was added in a concentration of 5 ng/µl.

HBOs were incubated with 50 µM propofol for 1 h at 80 div or for 3 days from 77 to 80 div. In total, 8 HBOs (4 treated with propofol, 4 treated with mock) were prepared for analysis from two independent culture batches and measured in duplicate in two separate experiments. For sample preparation, between 2 and 4 mg of the HBOs were weighed and homogenised. Subsequently, 10 µl of ISTD was added as for the calibrators. Extraction was performed for calibrators and HBOs by adding 100 µl hexane (VWR, Darmstadt, Germany) and intensive shaking for 5 min on a stirrer. The calibrators and samples were then centrifuged for 10 min at 15,000 rpm and the supernatant was transferred to vials. Extraction were not derivatized for further analysis with gaschromatography-mass spectrometry (GC-MS, Fig. S1).

Analysis was performed on an Agilent (Waldbronn, Germany) GC-MS system consisting of a 8890 Gaschromatograph (GC) coupled to a MSD 5977B single Quadrupole Mass Spectrometer (MS). Injector and transfer line temperatures were respectively 250 °C and 280 °C. The injection volume was 1 µl in splitless mode. Helium was used as a carrier gas, septum purge flow was 3 ml/min. The initial oven temperature of 60 °C was held for 3 min, followed by a temperature increase of 10 °C/min to a temperature of 120 °C, followed by another increase at a rate of 30 °C/min to a final temperature of 300 °C, which was held for 8 min. Total run time was 23 min for each sample. Electron impact ionisation (EI) was applied at 70 eV. Temperatures of the ion source and the quadrupole were set at 230 °C and 150 °C, respectively. For quantitation, the mass spectrometer was operated in selected ion monitoring (SIM) mode. A total dwell time of 120 ms was used. The m/z data for target and qualifier ions used for quantitation of propofol and propofol-D_17_ were 163.1, 178.1, 91.1 and 177.2, 195.2 and 98.1 respectively. The quantitative analysis was carried out with the software MassHunter Quantitative Analysis Version 12.1. Concentrations obtained were corrected for the weight of the HBOs.

### HBOs Growth and Size Measurement

For the HBO growth and size measurement, bright-field images of individual organoids were captured using an Evos microscope (AMG, Thermo Fisher) from 3 div to the end of the experiments. The two-dimensional cross-sectional area was analyzed using ImageJ software (RRID: SCR_003070) including the “Polygon selections” tool following the “Measure” plugin.

### Determination of Lactate Concentration

Lactate was measured in HBO medium using a L-Lactate Assay Kit according to the manufacturer`s instructions (MAK329, Sigma-Aldrich). A separate batch of HBOs was cultured as described above and individual HBOs were transferred at 47 div to 24 well plates, treated with propofol or mock (*n* = 4 each) from 47 div to 50 div, and cultured until 60 div. Lactate was determined in medium at 47 div as a baseline, at 50 div, and at 60 div, normalized to HBO weight, and expressed as mole/g.

### Immunofluorescence Staining

HBOs were collected at 60 div and washed once with ice cold phosphate-buffered saline (PBS) and fixed for 15 min in PBS containing 4% PFA, then washed three times in PBS, and transferred to 30% sucrose at 4 °C. Next day, specimens were embedded with Epredia Neg-50 Blue (Thermo Fisher Scientific) and cut to 12 μm cryosections using a cryotome (HM 560 Cryo-Star, Thermo Fisher Scientific). Sections were collected on Superfrost Plus Adhesion Microscope slides (New Erie Scientific LLC) and stored at −20 °C. For double-immunofluorescence staining, sections were air-dried at room temperature (RT) for 30 min, again fixed with 4% PFA for 10 min. After washing in PBS, sections were incubated with blocking solution (5% goat serum, 0.5% bovine serum albumin, 0.1% Triton X-100 in PBS) for 1 h at RT. Then, the sections were incubated with blocking solution containing primary antibodies specific for NeuN (dilution: 1:500, RRID: AB_2924930) or GFAP (dilution: 1:500, RRID: AB_10641162) at 4 °C overnight. The next day, the sections were washed with PBS and incubated with secondary antibodies (goat anti-rabbit Alexa 568, RRID: AB_2534121; goat anti-guinea pig Alexa 488, AB_2534117, dilution 1:1000 each) in blocking solution for 1.5 h at RT, washed in PBS, and counterstained with 4′,6-diamidino-2-phenyl-indol-dihydrochloride (DAPI, 1:10.000; Sigma‒Aldrich) for 5 min, washed in PBS and imaged using a fluorescence microscope (BZ-X800, Keyence) with appropriate filters settings using a 20x objective.

### High-Density MEA Recordings

Organoids at 60 div or 90 div were placed on Accura HD-MEA chips (3Brain, Switzerland) and anchored in place using a platinum anchor with mesh attached. Activity was recorded in the presence of DMEM at 37 °C. Spontaneous activity was observed in several channels, which was eliminated in presence of the specific voltage-gated sodium channel blocker Tetrodotoxin (TTX, 2 µM, Biozol), confirming that the HBOs demonstrate firing of action potentials. Activity was recorded with Brainwave 5 software (3Brain, Switzerland) for 5 min per condition, with a high-pass filter of 100 Hz, in dark conditions and with a Sampling Frequency of 19,754 Hz. The data files were exported, segmented and converted from analogue to digital voltage using code in Julia/Jupyter notebooks.

Timestamps of the re-calibration of the Accura HD-MEA chips, which induced artificial spikes in our recordings, were identified, and a border of 100 data points either side of the timestamp were eliminated, corresponding to about 10 ms every 400 ms. This procedure prevented that these re-calibration artefacts were included in the neuronal signal analysis. We also observed instances of “flashes” of activity, where the majority of channels were saturated. Those were also removed from the analysis. A bandpass Remez filter (150–3000 Hz) was applied to the data. Spike were detected as deviating by 5 x stds from the mean, within a 5 ms moving window to calculate the event threshold. We selected the 30 most active channels from the recording and compared the activity in these channels across the different experimental conditions and ensured that the average frequency of the channels were above a minimum activity level of 0.5 Hz (two organoids 90 div mock with an average frequency < 0.4 Hz were excluded).

### Bulk RNA-Sequencing

Total RNA was purified using RNAeasy Kit (Qiagen), the quantity was assessed with the Qubit 2.0 and quality was checked using a RNA 6000 Nano chip on Agilent’s bioanalyzer. Barcoded mRNA seq libraries were prepared from 400 ng total RNA using a NEBNext^®^ Poly(A) mRNA Magnetic Isolation Module and NEBNext^®^ Ultra™ II RNA Library Prep Kit for Illumina^®^ with a final amplification of 12 cycles. Library concentrations were quantified using Invitrogen’s Qubit HS DNA assay and average library size was analyzed on Agilent’s 2100 Bioanalyzer using a HS DNA chip. Sequencing was performed at Novogene (Cambridge, UK) on an Illumina NovaSeq 6000 sequencing system using a sequencing depth of 30 Mio paired end (150 cycles) reads per sample (150 cycles). Sample quality was assessed with demultiplexed fastq.gz files. Sequenced reads were trimmed for adapter sequences and processed using Qiagen’s software CLC Genomics workbench (v21.0.5) with CLC’s default settings for RNASeq analysis. Reads were aligned to GRCm38 genome (Waterston et al. 2002)using a minimum read length of 50 bp. The expression value unit is TPM. Results were displayed with ArrayStar 17 (Lasergene) including the number of mapped reads, target length, source length and position, strand, genes and gene IDs, annotated according to the mm10 assembly. Genes were filtered for at least 7 valid values out of 20 samples with normalized reads > 0.1 to exclude low expression genes.

Data were analyzed essentially as described (Wang et al. 2022). Briefly, differentially expressed genes (DEGs) were identified using log-transformed data, and Volcano plots were generated using GraphPad Prism to visualize DEGs, with the following criteria: absolute difference > 4, fold change (FC) > 2, and *p* < 0.05. Gene Ontology (GO) enrichment analysis of the selected DEGs was performed using the STRING database (https://string-db.org/*).* To assess functional implications based on all annotated transcripts, Gene Set Enrichment Analysis (GSEA) was conducted using the GSEA software (http://www.gsea-msigdb.org*)* (Subramanian et al. 2005) and showing enrichment plots of Gene Ontology Biological Process (GOBP). Heat maps were generated based on the genes associated with individual GO term using GraphPad Prism. Additionally, to investigate shared GO terms between two comparisons (60 div HBOs ± propofol, and 90 div HBOs ± propofol), Venn diagrams were generated using online tool (https://bioinformatics.psb.ugent.be/webtools/Venn/*)* and analysis was performed for all GOBP, GOCC, GOMF, and hallmark gene sets. GSEA ranked gene lists are based on fold difference and P value. RNA-seq data have been deposited as GEO dataset with the provisional accession number GSE294524: https://www.ncbi.nlm.nih.gov/geo/query/acc.cgi?acc=GSE294524.

### Statistical Methods

**Fig. 2:**
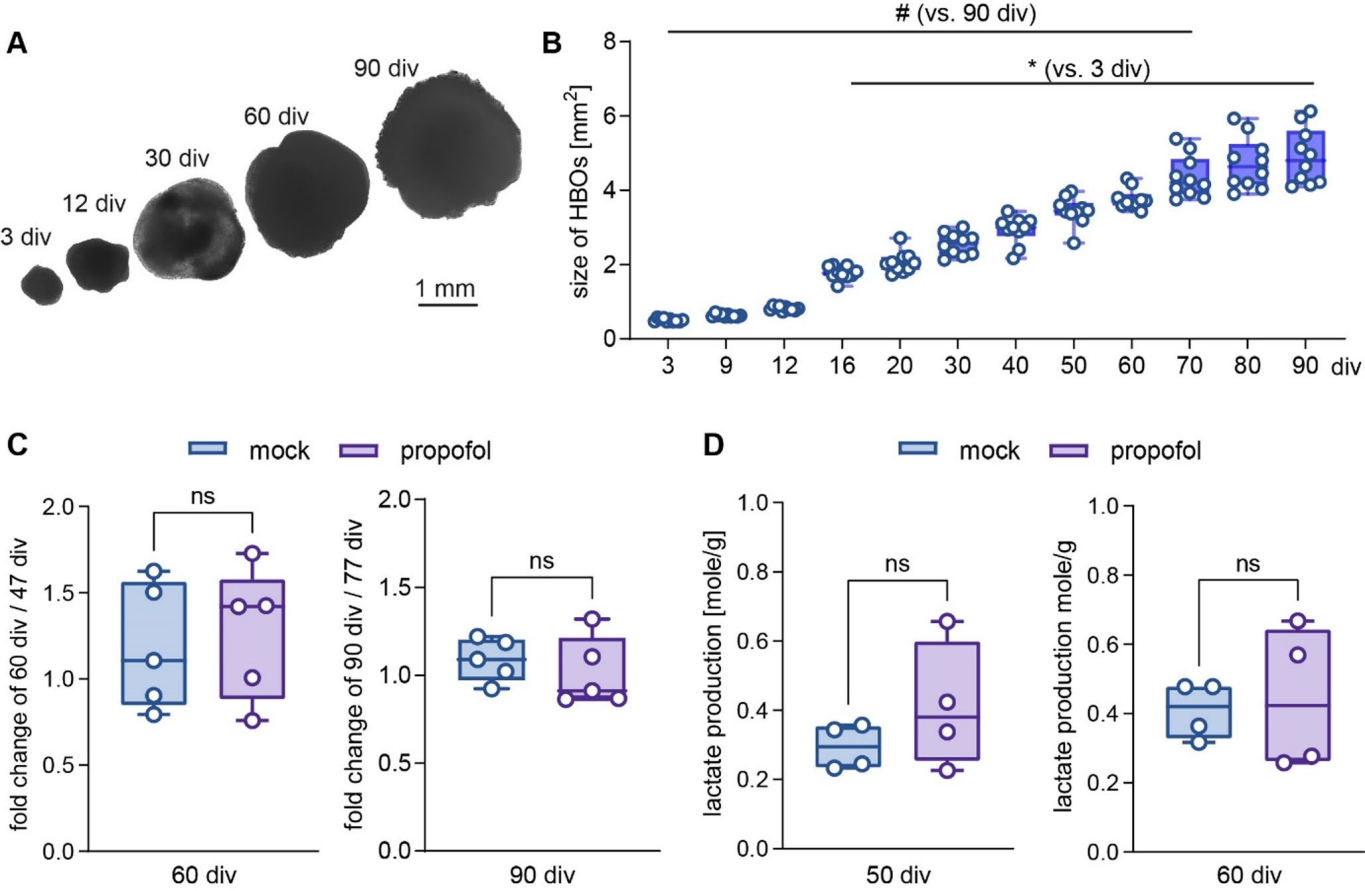
HBO growth rates and metabolism are not affected by intermittent exposure to propofol. **A** Bright field microscope images showing individual HBOs at different cultivation time points (scale: 1 mm). **B** HBOs size assessments from 3 div to 90 div demonstrated continuous growth (*n* = 10), with a significantly increased size from 16–90 div vs. 3 div (*p < 0.05) and a decreased size from 3–70 div vs. 90 div (#*p* < 0.05). Data were analyzed using One-Way-ANOVA followed by Šídák’s multiple comparison. **C** Fold change growth of HBOs at 60 div relative to 47 div, and 80 div relative to 77 div, following single treatment with mock or 50 µM propofol (*n* = 5 each). **D** Lactate production determined at 50 div and 60 div following mock- or propofol treatment from 47–50 div (normalized to HBO weights, *n* = 4 each). Data were analysed using Student`s unpaired two tailed t-test. Data are presented as box plots (median, 25th to 75th percentiles), and whiskers down to the minimum and up to the maximum value

**Fig. 3:**
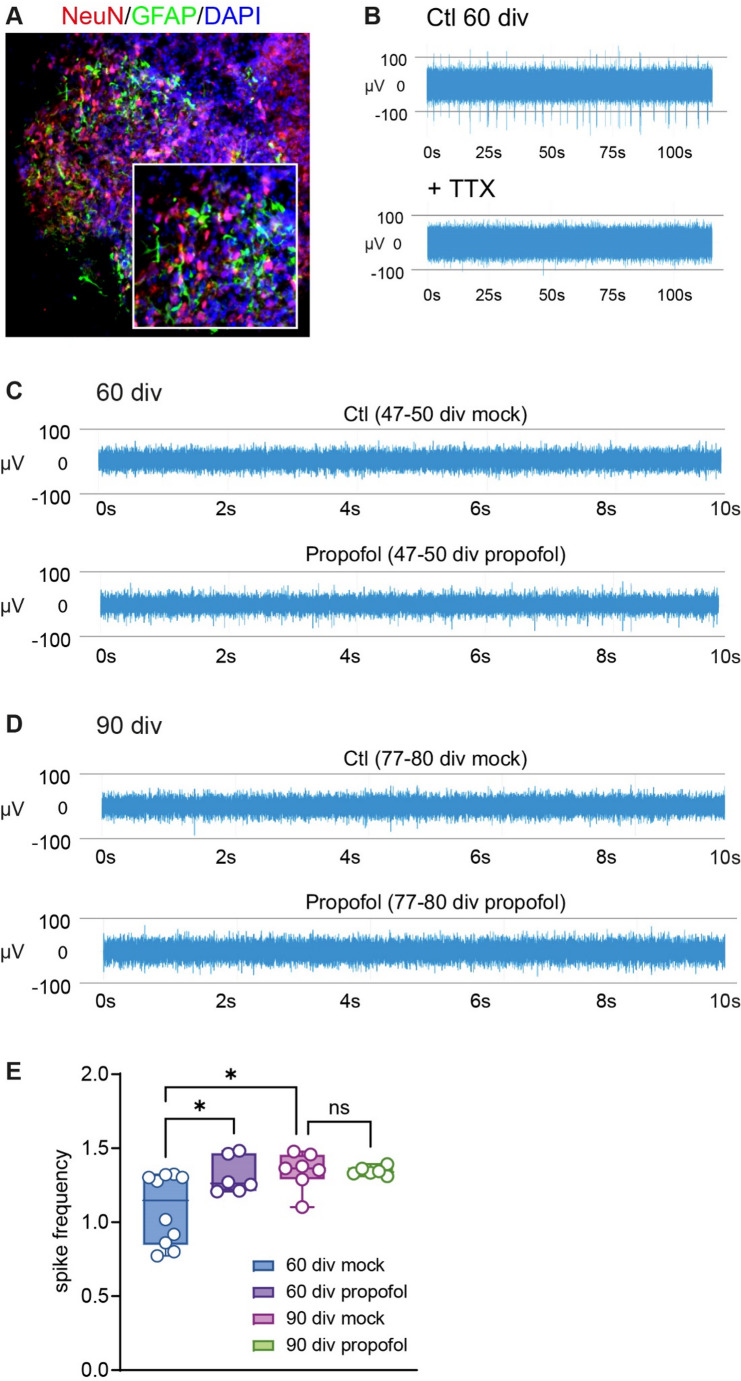
Intermittent propofol exposure to HBOs elicits developmental stage-specific effects on neuronal activity. **A** Immunofluorescence staining in 60 div HBO using anti-NeuN, anti-GFAP, and nuclear counterstaining (DAPI) indicating the presence of post-mitotic neurons and astrocytes. **B** HD-MEA recordings under control conditions and in presence of TTX at 60 div, demonstrating the neuronal original of spiking activity in HBOs. Note the missing multi-unit-activity (spikes) in the TTX-treatment condition **C**, **D** MEA recordings showing an increased spiking frequency in HBOs at 60 div (**C**), but not at 90 div (**D**), following IPE compared to mock treatment. **E** Summary graph of the spiking frequency in HBOs at 60 div and 90 div following IPE or mock treatment (*n* = 6–10/group), which show that propofol induced an increased spiking frequency at 60 div, an effect not observed at 90 div. *p < 0.05, One-Way-ANOVA followed by Šídák’s multiple comparisons

GraphPad Prism (version 10) was used for statistical calculation. Data are presented as box plots (median, 25th to 75th percentiles) with individual data points, and whiskers down to the minimum and up to the maximum value. To test for differences in HBO growth and lactate release (Fig. 2), pairwise comparisons of the parametrically distributed values were analysed using Student’s unpaired two-tailed t-test. To test for differences in spike frequencies of HBOs (Fig. 3), One-Way-ANOVA followed by Šídák’s multiple comparisons between the four experimental groups (60 div HBO ± propofol, 90 div ± propofol) was used to compare the top 30 channel recordings with the highest frequency. HBOs lacking activity (< 0.5 Hz mean activity of the active channels, *n* = 2, 90 div control group) or individual recording channels showing abnormally high activity across different HBOs (> 5 Hz, 0–3 channels per HBO) were excluded. Data were tested for statistically significant outliers using ROUT outlier test and for parametric distribution using Shapiro-Wilk test. *p* < 0.05 was considered statistically significant.

## Results

### Propofol Rapidly Accumulates at Supraclinical Concentrations in HBOs and Subsequently Decreases to Clinically Relevant Levels

Human brain tissue concentrations of propofol have been reported to reach up to 7.68 ng/mg (corresponds to 43 µM assuming water-like density) in patients undergoing resection of an epileptic focus (Van Hese et al. 2020). We determined the concentration of propofol in HBOs following administration of propofol at 50 µM to the culture medium (corresponds to 8.9 µg/ml), using GC-MS. This analysis revealed an initial propofol concentration 172.9 ± 43 ng/mg (mean ± SD) after 1 h, and a concentration of 6.1 ± 1.2 ng/mg (mean ± SD) in HBOs after 72 h of propofol exposure. Thus, propofol exposure resulted in a rapid accumulation at supraclinical concentrations in HBOs, whereas prolonged exposure resulted in a decrease to clinically relevant levels.

### IPE Does Not Influence HBO Growth Rates or Lactate Metabolism

HBOs exhibited continuous growth until the final observation time point at 90 div under mock conditions, as assessed through microscopic image analysis (Fig. 2A, B). Next, possible growth disturbances from IPE were examined (Fig. 2C). Sizes of HBOs exposed to propofol (50 µM) or mock from 47 to 50 div were determined from 47 div to 60 div, and those treated from 77 to 80 div were measured from 77 div to 90 div. This enabled us to calculate the fold change of HBO growth over time, which, however, did not reveal differences between propofol and mock treatment (Figs. 2C and 47–60 div: mock 1.19 ± 0.16, propofol 1.27 ± 0.17, *p* = 0.738; 77–90 div: mock 1.09 ± 0.05, propofol 1.01 ± 0.09, *p* = 0.492, unpaired t test).

HBOs exhibit high glycolytic activity and lactate release (Castilla Bolanos et al. 2024), and propofol was reported to inhibit glycolysis and lactate release in acute rat brain slices (Hadjihambi et al. 2020). Therefore, we compared lactate release from HBOs following IPE or mock treatment from 47 to 50 div (Fig. 2D). However, lactate concentrations determined in the cell culture medium at 50 div or 60 div did not differ significantly between the experimental groups (Figs. 2D and 50 div: propofol 0.41 ± 0.09 SEM, mock 0.29 ± 0.03 SEM, *p* = 0.274; 60 div: propofol 0.44 ± 0.1 SEM, mock 0.4 ± 0.04 SEM, *p* = 0.769; unpaired t test). These results indicated normal growth and metabolism and the absence of obvious neurotoxicity (i.e. an irreversibly catastrophic metabolic failure) in HBOs following IPE.

### IPE to HBOs Induces Developmental Stage-Specific Effects on Neuronal Activity

At 60 div, double-immunofluorescence staining identified cells expressing the astrocyte marker GFAP, and the mature neuron marker NeuN (Fig. 3A), indicating that HBOs exhibited a maturation stage with neuronal activity (Fair et al. 2020; Trujillo et al. 2019). MEA recordings were conducted to confirm electrophysiological activity in HBOs. Indeed, the HBOs exhibited multi-unit activity in the MEA-recording indicating spiking activity, i.e. spontaneous firing of action potentials. This activity was eliminated in the presence of TTX, a selective blocker of voltage-dependent sodium channels (Fig. 3B), confirming firing of neuronal action potentials in HBOs.

To examine developmental maturation and possible stage-specific effects of propofol on neuronal activity, a total of 32 HBOs were recorded (Fig. 1, *n* = 16 at 60 div, *n* = 16 at 90 div, equal group size for propofol/mock treatment). The vast majority of HBOs in the experimental groups exhibited spiking activity (93.75% success rate, two HBOs of 90 div mock treatment group lacked activity) (Fig. 2C, D). Analysis and multiple comparisons of spike frequency between the four experimental groups revealed an increase from 60 div to 90 div in the absence of propofol treatment, consistent with developmental maturation in HBOs (Fig. 3E). At 60 div, IPE from 47 to 50 div caused a significantly increased spiking frequency in HBOs compared to mock treatment. At 90 div, spiking frequency in HBOs with IPE from 77 to 80 div was not different compared to mock treatment (Fig. 3E). These findings indicated that IPE resulted in a sustained increase in neuronal activity in HBOs at 60 div, but not at 90 div, suggesting a developmental stage-specific mode of action.

### IPE Selectively Increased the Expression of Developmentally Regulated Genes in HBOs

The findings from MEA recordings suggested that IPE alters neurodevelopmental maturation of HBOs. Therefore, we conducted bulk RNA-sequencing and subsequent data analysis to compare transcriptome profiles of HBOs at 60 div and 90 div following IPE or mock (Fig. 1).

**Fig. 4:**
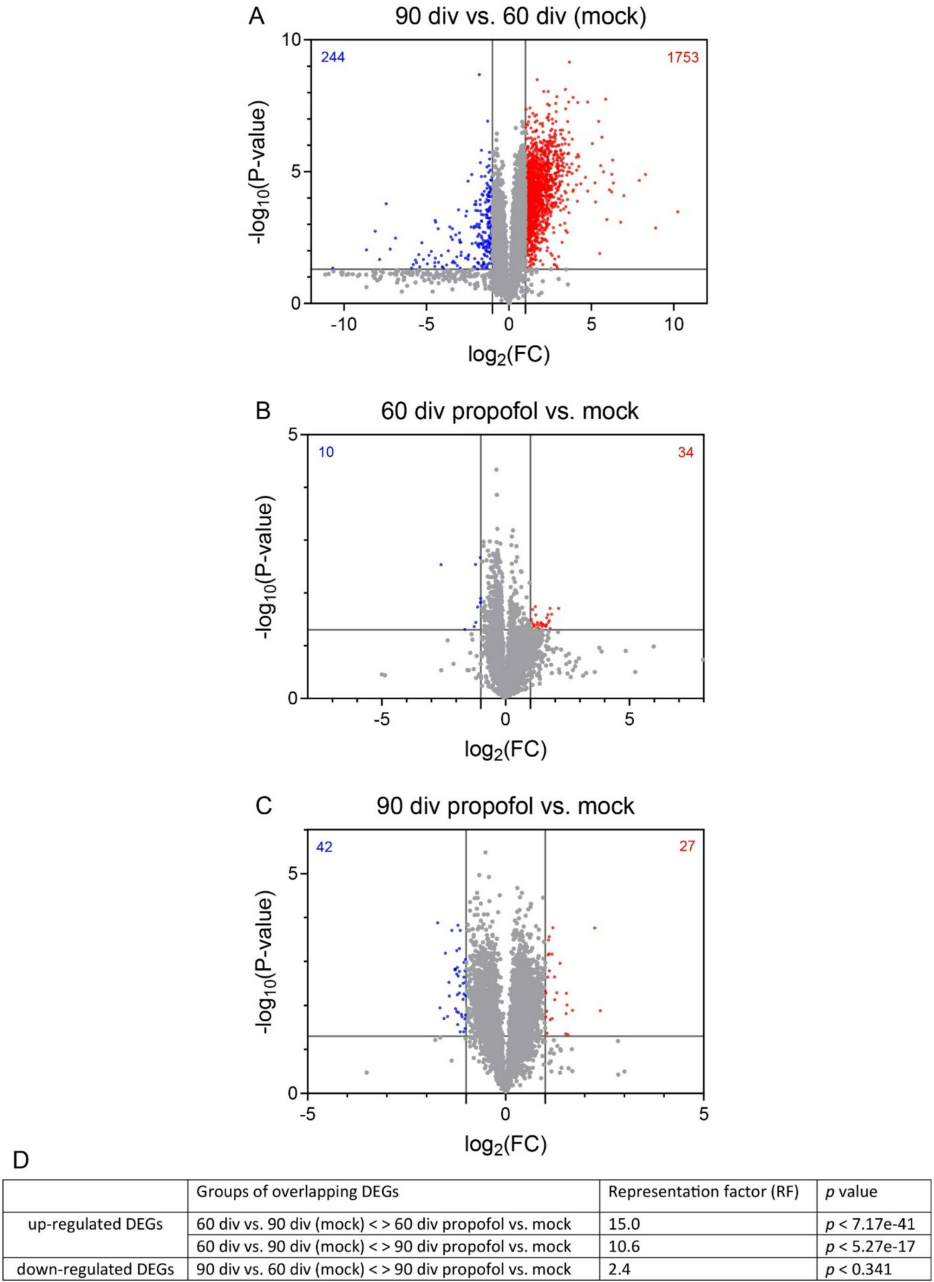
Intermittent propofol exposure selectively enhances the expression of developmentally regulated genes in HBOs. **A**–**C** Volcano plots show DEGs (criterion: absolute difference > 4, fold change > 2, *p* < 0.05) in mock treatment 90 div vs. mock treatment 60 div, mock treatment 60 div vs. propofol treatment 60 div, and mock treatment 90 div vs. propofol treatment 90 div, respectively (*n* = 5). The X-axis represents the log_2_-transformed fold change (log_2_ FC) and the Y-axis represents the negative log_10_-transformed p-value. Significantly upregulated (red dots) or downregulated (blue dots) DEGs and non-significantly regulated genes (grey dots) are shown. **D** Representation factors and p values calculated for up- and down-regulated DEGs overlapping across experimental groups and developmental stages (http://nemates.org/MA/progs/overlap_stats.html) (Cho et al. 2021)

First, we revealed DEGs following stringent data sorting (absolute difference > 4, fold change > 2, *p* < 0.05) between 60 div and 90 div HBOs, anticipating a substantial number of developmentally up-regulated genes. As expected, this analysis revealed 1997 DEGs with 1753 genes up-regulated and 244 genes down-regulated in HBOs at 90 div compared to 60 div (Fig. 4A).

We next compared HBOs at 60 div, with or without IPE from 47 to 50 div, using identical data sorting criteria. This resulted in 44 DEGs with 34 genes up-regulated and 10 genes down-regulated in HBOs treated with propofol (Fig. 4B). Notably, all genes upregulated in 60 div HBOs in response to IPE were also upregulated in 90 div vs. 60 div (mock) HBOs. Conversely, 2 of out 10 DEGs downregulated in HBOs following propofol exposure were also downregulated in mock-treated HBOs at 90 div vs. 60 div (Fig. 4C).

To assess the significance of overlapping DEGs between groups, a representation factor (RF, http://nemates.org/MA/progs/overlap_stats.html) (Cho et al. 2021) was calculated (Fig. 4D). This calculation revealed a particularly significant overlap between up-regulated DEGs of the comparison 60 div versus 90 div and up-regulated DEGs of the comparison propofol 60 div versus mock 60 div (RF = 15.0, *p* < 7.17e-41). Comparison of HBOs at 90 div, with or without IPE from 77 to 80 div, revealed 69 DEGs with 27 genes up-regulated and 42 genes down-regulated in HBOs treated with propofol. Again, 19 out of 27 genes up-regulated following propofol treatment from 77 to 80 div were also up-regulated in mock-treated HBOs at 90 div compared to 60 div (RF = 10.6, *p* < 5.27e-17). In contrast, only 1 out of 42 genes was down-regulated both in HBOs at 90 div following propofol treatment from 77 to 80 div compared to non-treated HBOs and in HBOs at 90 div vs. 60 div without propofol exposure (RF = 2.4, *p* < 0.341). These results demonstrated that IPE selectively increased the expression of genes developmentally regulated from 60 div to 90 div in HBOs.

### IPE to HBOs Results in Stage-Specific GO Enrichment of DEGs

**Fig. 5:**
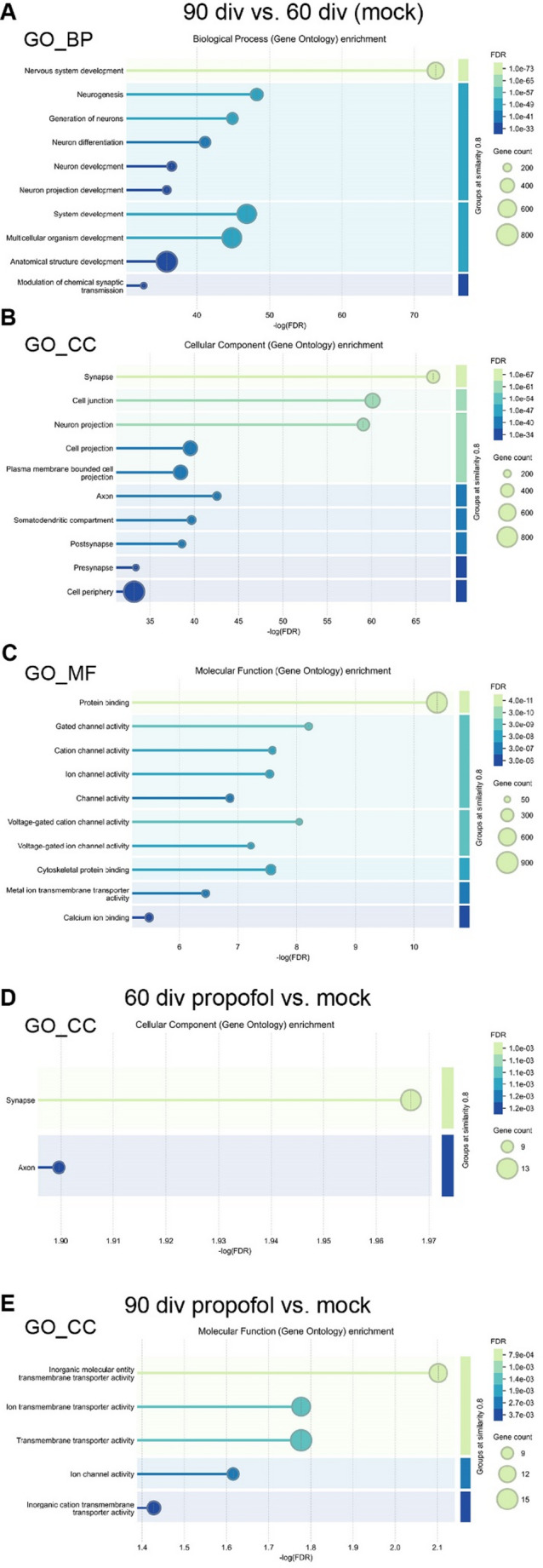
Propofol-induced DEGs are enriched in neurodevelopment and synapse-associated GO terms. **A**–**E** GO enrichment analyses were performed using STRING-DB, revealing significantly enriched GO terms. The X-axis represents the negative log10-transformed statistical significance of enrichment as -log_10_ (FDR), with larger values indicating stronger significance. Each circle represents a GO terms, with circle size proportional to the gene count associated with that term, and colour indicating the FDR as shown in the legend on the right. The intensity cut off for grouping was set at 0.8

Gene ontology (GO) enrichment analysis of DEGs was conducted with STRING DB (https://string-db.org/), separately for GO enrichment in the categories biological process (GO_BP), cellular component (GO_CC), and molecular function (GO_MF) (Fig. 5A-E, permalinks are provided as supplementary material). The comparison of HBOs at 90 div vs. 60 div demonstrated the association of DEGs with various GO terms of the categories GO_BP, GO_CC, and GO_MF related to neuronal development such as nervous system development, neurogenesis, and neuron differentiation, for example (Fig. 5A-C).

As mentioned before, the comparison between HBOs intermittently exposed to propofol or mock revealed a relative small number at 60 div (44 DEGs) or at 90 div (69 DEGs). As a consequence, GO enrichment of DEGs were not found in all categories. However, at 60 div, the comparison between IPE and mock treatment revealed the association of DEGs with the GO_CC terms “synapse” and “axon” (Fig. 5D). At 90 div, in contrast to 60 div, DEGs associated with GO_MF terms such as “transmembrane transporter activity” and “ion channel activity” (Fig. 5E).

### GSEA Reveals Opposing Developmental Stage-Specific Effects of IPE

The low number of DEGs in HBOs intermittently treated with propofol as compared to mock did not reveal GOBP, GOMF, and/or GOCC enrichments using STRING-DB. We therefore used GSEA which calculates enrichments based on all annotated transcripts, thereby providing an expanded overview of GO associations of BP, MF and CC as well as marker genes (Subramanian et al. 2005).

**Fig. 6:**
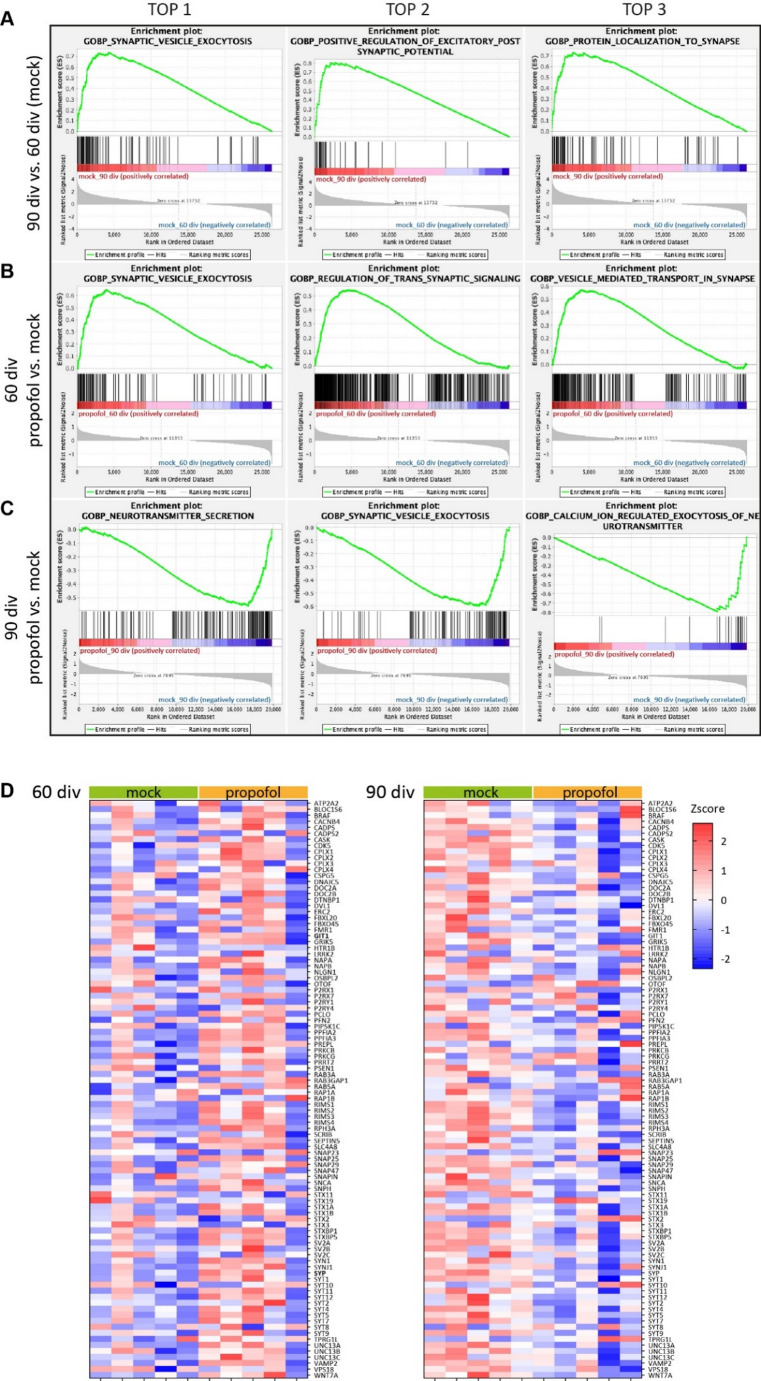
GSEA reveals opposing developmental stage-specific effects of propofol.**A**–**C** Enrichment plots, generated using GSEA 4.1.0, showing the top 3 GOBPs associations for the group comparisons of **A** 90 div vs. 60 div (mock), **B** 60 div propofol vs. mock, and **C** 90 div propofol vs. mock. The green line chart indicates the gene enrichment score (ES) and the peak represents maximum enrichment. The vertical black bars indicate single genes belonging to the gene set. The bottom part shows the change in all genes, red indicates high expression and blue indicates low expression. **D** Heatmaps of individual genes expressed in HBOs belonging to the GOBP_Synaptic_Vesicle_Exocytosis (GO: 0016079) for the comparisons of 60 div propofol vs. mock, and 90 div propofol vs. mock. Relative expression levels (Zscore) of 96 genes are shown, red to blue colour represents high to low expression

GSEA revealed that IPE from 47 div to 50 div resulted at 60 div in increased associations with various GOBP terms of neurodevelopment and synapses such as synaptic vesicle exocytosis (Fig. 6A-C), regulation of axonogenesis, dendrite development, and synapse assembly (Fig. S2), whereas rather opposing effects were observed at 90 div following propofol treatment from 77 div to 80 div (Fig. 6A-C, Fig. S2). In contrast, GSEA did not reveal significant differences in the “Hallmark_Apoptosis” gene set, supporting the lack of obvious neurotoxic effects of propofol (Fig. S2).

The top 3 GOBP terms for each comparison between experimental groups (60 div vs. 90 div (mock), 60 div propofol vs. mock, 90 div propofol vs. mock) were related to synaptic function as demonstrated by GSEA enrichment plots (Fig. 6A-C) (according to FDR and *p* value). The enrichment scores of genes up-regulated from 60 div to 90 div were slightly higher, but similar to the enrichment scores calculated between 60 div propofol vs. mock (Fig. 6A, B). However, opposing effects were observed for the comparison between 90 div propofol vs. mock (Fig. 6C). Notably, the GOBP_Synaptic_Vesicle_Exocytosis (GO: 0016079) was among the top 3 GOBPs in all comparisons between experimental groups (Fig. 6A). Consistent with the developmental stage-specific effects of propofol on neuronal activity and DEGs, heatmaps of individual genes expressed in HBOs belonging to the GOBP_Synaptic_Vesicle_Exocytosis (Fig. 6B) demonstrated overall opposing regulations at developmental stages 60 div and 90 div by IPE compared to mock treatment (expressed as Zscores and log2FC, Fig. S3).

**Fig. 7:**
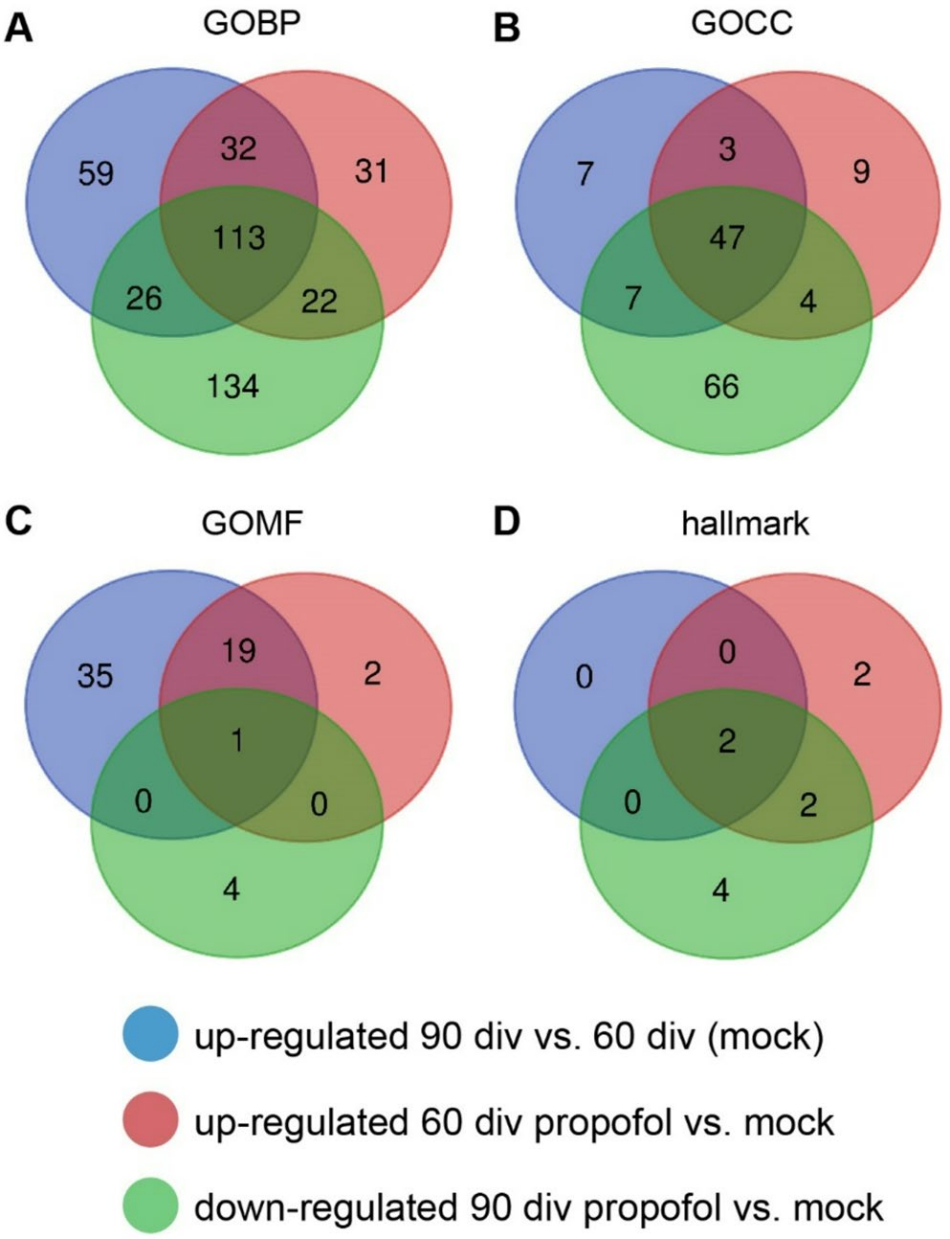
Propofol induces developmental stage-specific gene set regulations. VENN diagrams of significantly regulated GOBP, GOCC, GOMF, and hallmark gene sets (criteria: FDR value < 0.05, normalized p value < 0.05), with blue circle representing upregulation of mock treatment 90 div versus mock treatment 60 div, red circle representing upregulation of propofol treatment 60 div versus mock treatment 60 div, and green circle representing downregulation of propofol treatment 90 div versus mock treatment 90 div. The number of shared gene sets associated with the propofol treatment at each developmental stage are shown in the overlapping regions

Next, we tested whether the opposing developmental stage-specific effects of propofol are visible across various gene sets in GO terms of different categories (Fig. 7). We generated VENN diagrams of significantly regulated GO terms (GOBP, GOCC, GOMF, FDR value < 0.05, p value < 0.05) both upregulated at 60 div HBOs + propofol and downregulated in 90 div HBOs + propofol. This analysis showed unique or shared GO terms associated with the propofol treatment at each developmental stage. In particular, 135 GOBPs, 51 GOCCs, 1 GOMF, and 4 hallmark GOs were found to be oppositely regulated by propofol depending on the developmental stage (Fig. 7). Hence, propofol induced developmental stage-specific alterations across various gene sets in GO terms of different categories.

## Discussion

Neuronal activity is a critical determinant in the development of neuronal structures, neuronal differentiation and synaptic connectivity. For example, transient alterations in neuronal activity during critical stages of brain development affect network connectivity and result in cognitive symptoms related to neuropsychiatric disorders (Bitzenhofer et al. 2021; Kirischuk et al. 2017; Marín 2016). Consequently, anaesthetic suppression of neuronal activity during pregnancy potentially affects neuronal differentiation and synaptic connectivity of the fetal brain (Rizzi et al. 2008).

Various studies have demonstrated that HBOs are suitable for modeling fetal human brain development in health and disease (Bai 2024; Coronel et al. 2026; Logan et al. 2020; Sidhaye and Knoblich 2021). In the present study, IPE to HBOs from 47 to 50 div resulted in increased neuronal activity at 60 div, an effect not observed at 90 div, following exposure from 77 to 80 div. RNA-seq has identified developmental stage-specific transcriptomic profiles with DEGs that reflect neuronal differentiation and synapse formation from 60 to 90 div. Notably, an identical subset of DEGs associated with synapse function was observed at 60 div following IPE, suggesting accelerated maturation. In contrast, synapse function-associated DEGs were down-regulated following IPE at 90 div, indicating stage-specific effects of propofol during HBO maturation. While the increased neuronal activity at 60 div in propofol-treated HBOs, as evidenced by MEA recordings, was consistent with increased expression of synapse-associated genes at the same time-point, this functional correlation was not observed at 90 div. Further studies are required including immunofluorescence analyses of neuronal maturation and subpopulation markers, as well as pre- and post-synaptic proteins, are needed in future studies. It is a limitation of our study that gene expression changes were not validated at the protein level.

Our intermittent treatment regime using single administration of 50 µM propofol to the cell culture medium was chosen based on clinical human brain tissue concentrations reaching up to 43 µM (Van Hese et al. 2020) and the absence of toxic effects in iPSC-derived NPCs after a 6-hour exposure to 50 µM propofol (Long et al. 2017). One hour after administration we observed supraclinical concentration, probably due to propofol`s high lipid solubility, which then decreased 72 h after administration to clinically relevant concentrations. However, it is important to consider that slow equilibration rates attributable to the lipophilicity of propofol, as previously demonstrated in rat brain slice cultures (Gredell et al. 2004), may have resulted in concentration gradients. Consequently, neurons situated near the surface of the HBO may have been exposed to higher concentrations than those located deeper. However, the formation of such concentration gradients within our HBO model and their potential impact on neuronal activity and the transcriptomes of HBOs cultured for different durations have not been investigated in this study.

The decline of propofol concentrations from 1 h to 72 h after administration suggested the existence of metabolic pathways degrading propofol in HBOs. Human gene variants in cytochrome P450 (CYP) and UDP-glucuronosyltransferase (UGT) have been linked to adverse clinical outcomes such as the propofol-related infusion syndrome (Budic et al. 2022; Dinis-Oliveira 2018). Among the 57 genes encoding for CYPs, 12 members of CYP family were expressed at levels considered as relevant (absolute expression value > 3). At 90 div, 16 CYP genes had an absolute expression value > 3. In contrast, among the 22 genes encoding for UGTs, only UGT8 was expressed at relevant levels in HBOs at 60 div and 90 div. Based on these expression data, it seems possible that metabolic pathways involving CYPs may have contributed to a decrease in propofol levels.

A fast equilibration between plasma and brain tissue has been assumed due to propofol’s high lipid solubility (Sahinovic et al. 2018), with venous blood concentration mother-to-fetus ratio ranging from 0.7 to 0.8 (Dailland et al. 1989; Sahinovic et al. 2018). To our knowledge, data regarding propofol concentrations in the fetal brain during the administration of sedative doses to the mother over extended periods have not been reported yet and it remains unclear to what extent the propofol concentrations in HBOs in the present study are comparable with those in clinical settings. To answer this question, additional studies on dose-response relationships are necessary by using a wide range of propofol concentrations and varying the duration of treatment as well as the observation time points after treatment, along with data on clinical concentrations in fetal brain tissue.

Nonetheless, initially high concentrations of propofol in HBOs may have elicited neurotoxic effects. Indeed, transient neurotoxic effects of propofol have been observed in neonatal rats as evidenced by transiently increased levels of activated caspase-3, down-regulation of neurotrophic factors and synaptic proteins (Chen et al. 2016; Karen et al. 2013; Milanovic et al. 2010; Pešić et al. 2015) as well as region-specific synapse formation or elimination (Karen et al. 2013; Milanovic et al. 2010; Pešić et al. 2015). At the same time, the activation of compensatory mechanisms (i.e. up-regulation of GAP-43 and MAP2) in response to propofol-induced neurotoxicity has been observed (Milanovic et al. 2017). Together, these findings demonstrated that propofol can induce immediate changes in activity-dependent processes, synaptic function, and plasticity. Consequently, it is possible that the increased neuronal activity of HBOs at 60 div resulted from initial transient neurotoxic effects of IPE, followed by processes related to plasticity.

To obtain a comprehensive understanding of the processes during and subsequent to IPE, it would be necessary to assess both specific markers of apoptosis, oxidative stress, and mitochondrial dysfunction (i.e. cleaved caspase 3, reactive oxygen species, and mitochondrial membrane potential) and neuronal maturation and plasticity at time points during propofol treatment from 47 to 50 div and 77–80 div, as well as at subsequent post-treatment intervals until the 60 div and 90 div endpoints.

Previous studies using primary neuronal cultures, or human iPSC-derived NPCs, showed neurotoxic effects or impairment of mitochondrial function following administration of propofol at concentrations ranging from 10 to 100 µM or 20–300 µM, respectively (Berndt et al. 2018; Long et al. 2017). Recently, a 6-hour treatment of human fetal prefrontal cortex cultures with 20 µM propofol was found to cause transient changes in single-cell transcriptome profiles across various cell types including excitatory and inhibitory neurons, astrocytes, oligodendrocyte progenitor cells, and microglia. However, these changes did not alter single-cell trajectories and the removal of propofol reversed the effects (Chang et al. 2023). Whether propofol treatment leads to longer-lasting effects remains unresolved. Our findings based on extended treatment duration, indeed suggest that such lasting effects may occur. In brain organoids, a culture medium concentration of 35.6 ng/ml (200 µM) caused cell death as indicated by TUNEL staining (Saglam-Metiner et al. 2024). However, we did not observe obvious neurotoxic effects of IPE, as assessed by HBO size, growth rates, and release of the metabolic marker lactate. Given that lactate release can serve as an indicator of mitochondrial function in HBOs (Cho et al. 2021), these results further suggest that propofol did not lead to a sustained inhibition of energy metabolism.

In addition, RNA-seq data analysis concentrating on the GO term “regulation of neuron apoptotic process” (GO:0043523) did not reveal a characteristic signature of widespread neuronal apoptosis (supplementary material). However, DEGs between IPE and mock were observed for this GO term at 60 div (18 out of 326) and 90 div (102 out 0f 326), suggesting a stage-dependent, adaptive stress response.

Nonetheless, in addition to the already mentioned requirement for conducting immunofluorescence analyses of neuronal markers, a limitation of our study is the lack of examination of markers for activated caspase-3, reactive oxygen species, and mitochondrial membrane potential, which have been previously associated with neurotoxic effects of propofol (Liang et al. 2022; Milanovic et al. 2010, 2016).

Propofol’s primary mode of action during acute treatment is the slowing of GABA_A_ receptor channel closing, thereby potentiating inhibitory activity and reducing of neuronal firing (Bai et al., 1999) However, our experiments addressed the long-lasting effect on neural development and it should be noted that our treatment regime comprised a “two phase approach”, initially involving exposure to propofol for 3 div to simulate the effects of prolonged administration of propofol, followed by 10 div without propofol. Consequently, the alterations in neuronal activity and gene expression profiles result from a combination of effects occurring during propofol exposure and subsequent adaptive changes in the absence of the drug. The results obtained from RNA-sequencing indicate that propofol enhances HBO maturation. This hypothesis is supported by the significant overlap of DEGs associated with synapse functions observed between propofol and control conditions at 60 div, as well as between 60 div and 90 div in the absence of propofol. In agreement with these findings, earlier studies suggested that propofol promotes neuronal differentiation of neural stem cells (NSC) in vitro (Sall et al. 2012). Moreover, we observed increased spontaneous spike activity following IPE at 60 div. Previous studies demonstrated the maturation of neuronal networks at comparable periods in HBOs (Fair et al. 2020; Zafeiriou et al. 2020). Thus, we propose that IPE to HBOs subsequently enhanced neuronal maturation and it is a significant finding of this study that this effects was confined to an early developmental period and was not observed at a more advanced maturation stage of HBOs.

This raises question on the impact of propofol (and other anaesthetics) during critical developmental periods, excitation/inhibition balance, including the developmental shift of depolarizing to hyperpolarizing actions of GABA, and homeostatic plasticity. Indeed, a rapid increase in dendritic spine density was reported following propofol administration in young mice (De Roo et al. 2009) and has been associated with the developmental GABA shift (Puskarjov et al. 2017). The GABA shift is crucial for GABAergic synapse formation and neural circuit development (Ben-Ari et al. 2012; Chudotvorova et al. 2005) and has been shown to occur approximately at 40 div in human iPSC-derived bioengineered neuronal organoids (BENOs)(Zafeiriou et al. 2020). Given propofol`s function as a GABA_A_ receptor positive allosteric modulator (Olsen 2018), it may modulate this process when exposed to HBOs from 47 to 50 div, resulting in increased neuronal activity and synapse-associated gene expression at 60 div. Patch-clamp recordings of neuronal responses to GABA and propofol in HBOs at different developmental stages will be necessary to test this hypothesis in future studies. The stage-specific effects of propofol observed in this study further underscore the necessity to carefully consider the maturation state of HBOs in future developmental, neurotoxicity, or compound screening studies.

In general, use of an HBO model in combination with 2D HD-MEA recordings allows to sufficiently access the electrophysiological parameters of the tissue. However, limited contact areas of a 2D MEA might be circumvented using 3D MEA systems (Huang et al. 2022; Sharf et al. 2022). In combination with HBO slices this would allow to increase the number of neurons contacting the electrodes (Sharf et al. 2022). The 90 div HBOs developed necrotic cores probably due to diffusion limits within the organoid, as reported (Sharf et al. 2022). This may interfere with functional properties of HBOs and may explain the absence of propofol effects in 90 div HBOs. More advanced complex HBO models, including vascularized and mixed lineage tissue from PSCs (Adlakha 2023), should be employed to investigate the effects of propofol. Recently, a “ICU patient-on-a-chip model” was used to study effects of a 4-days propofol administration on the blood-brain barrier, mast cells, and cerebral organoids. Although this study did not explore consequences of propofol on neuronal activity, it suggested an association between increased expression of pro-inflammatory cytokines and neurotransmitter receptor subunits (Saglam-Metiner et al. 2024).

In conclusion, we identified stage-specific effects of IPE on neuronal activity and maturation in HBOs, thereby providing new insights on anaesthetic mechanisms during critical developmental periods. Future research should focus on more advanced HBO models, exploring mechanisms underlying stage-specific effects, and their long-term consequences, which may contribute to improving anaesthetic safety during pregnancy and early life.

## Supplementary Information

Below is the link to the electronic supplementary material.

Supplementary material 1 (DOCX 784.3 kb)

Supplementary material 2 (DOCX 967.4 kb)

## Data Availability

RNA-seq data have been deposited as GEO dataset with the provisional accession number GSE294524: https://www.ncbi.nlm.nih.gov/geo/query/acc.cgi? acc=GSE294524.
