# Supplementary material for: Intermittent Propofol Exposure Induces Neurodevelopmental Alterations in Human Brain Organoids
Source: Cell Mol Neurobiol. 2026 Jan 26;46:37. doi: 10.1007/s10571-026-01673-2 (PMC12901785; doi:10.1007/s10571-026-01673-2)
Supplement: Supplementary file 1 — Supplementary material 1 (DOCX 784.3 kb) [file 10571_2026_1673_MOESM1_ESM.docx]

# **Supplementary material**

## **Table 1**

Neural induction (NI) medium:

| DMEM/F12 | Gibco |
| --- | --- |
| 1 % (v/v) N2 | Gibco |
| 1 % (v/v) GlutaMax supplement | Gibco |
| 1 % (v/v) MEM-NEAA | Gibco |
| 1µg/ml Heparin | Sigma-Aldrich |

Differentiation medium without vitamin A (DM - A):

| DMEM/F12 : Neurobasal medium 1:1 | Gibco |
| --- | --- |
| 0.5 % (v/v) N2 supplement | Gibco |
| 1 % (v/v) B-27 supplement without vitamin A | Gibco |
| 1 % (v/v) GlutaMax supplement | Gibco |
| 0.5 % (v/v) MEM-NEAA solution | Gibco |
| 2.5 µg/ml Insulin | Sigma-Aldrich |
| 3.5 µl/L 2-Mercaptoethanol | Sigma-Aldrich |
| 1 % (v/v) Penicillin / Streptomycin | Gibco |

Differentiation medium with vitamin A (DM + A):

| DMEM/F12 : Neurobasal medium 1:1 | Gibco |
| --- | --- |
| 0.5 % (v/v) N2 supplement | Gibco |
| 1 % (v/v) B-27 supplement with vitamin A | Gibco |
| 1 % (v/v) GlutaMax supplement | Gibco |
| 0.5 % (v/v) MEM-NEAA solution | Gibco |
| 2.5 µg/ml Insulin | Sigma-Aldrich |
| 3.5 µl/L 2-Mercaptoethanol | Sigma-Aldrich |
| 1 % (v/v) Penicillin / Streptomycin | Gibco |

## **Figure S1:**


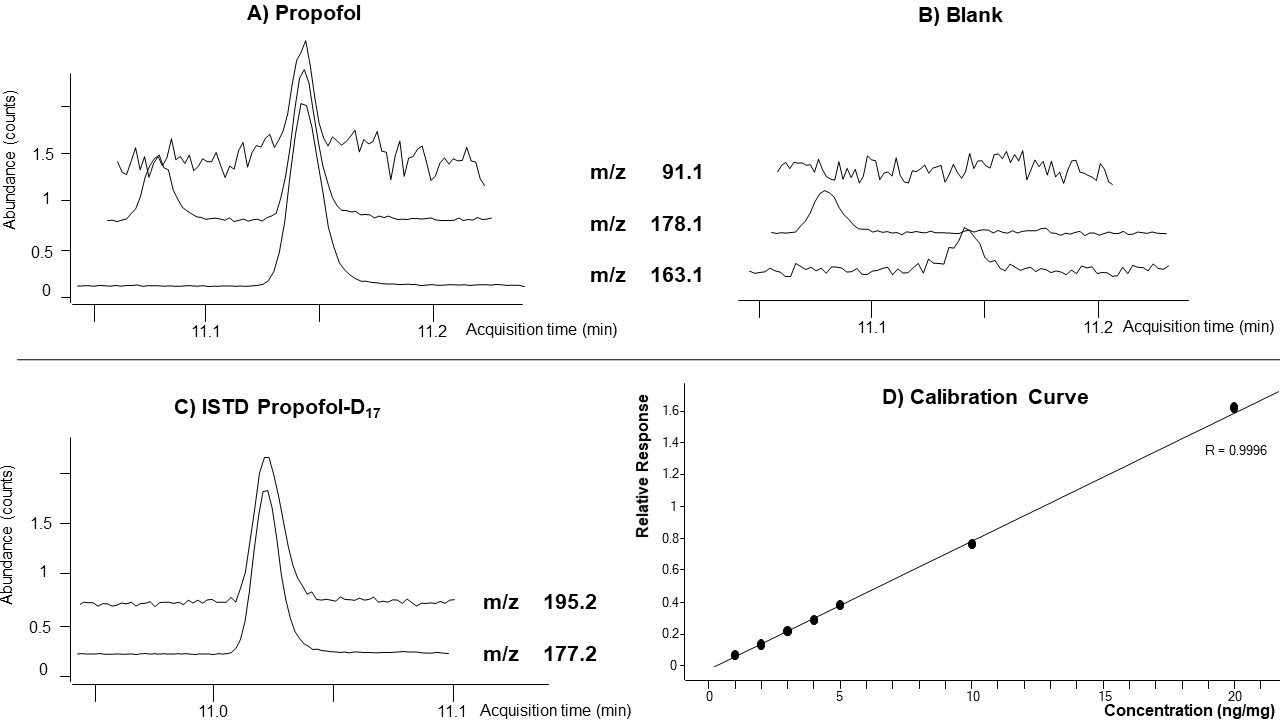


**Fig. S1: Analysis of propofol concentrations in HBOs**. A) Mass spectrometric signal of propofol with the ion traces m/z 163.1, 178.1 and 91.1 of an HBO sample after 3 days of incubation with propofol. B) Ion traces of the corresponding blank sample (human brain). C) Ion traces m/z 177.2 and 195.2 of the internal standard (ISTD) Propofol-D_17_. D) Corresponding calibration curve in the matrix human brain (concentrations 1-20 ng/mg) with a linearity R > 0.99.

## **Figure S2:**


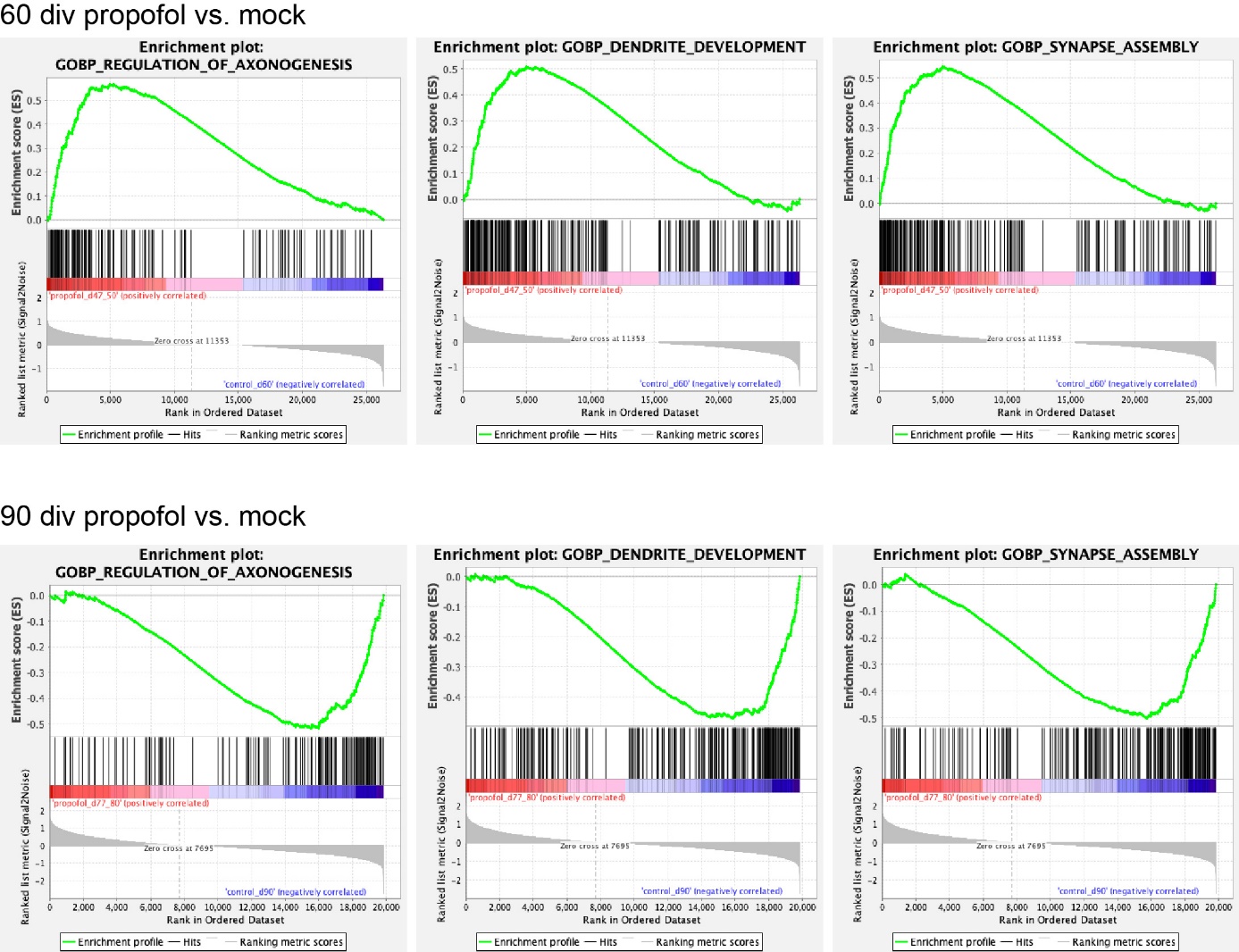


**Fig. S2: Enrichment plots.** GOBPs associations for 60 div propofol vs. mock (upper panel), and 90 div propofol vs. mock (lower panel) are shown. The green line chart indicates the gene enrichment score (ES) and the peak represents maximum enrichment. The vertical black bars indicate single genes belonging to the gene set. The bottom part shows the change in all genes, red indicates high expression and blue indicates low expression. GOBP terms regulation of axonogenesis, dendrite development, and synapse assembly were positively correlated with HBOs at 60 div following treatment with propofol from 47-50 div, whereas rather opposing effects were observed at 90 div following propofol treatment from 77 div to 80 div.

## **Figure S3:**


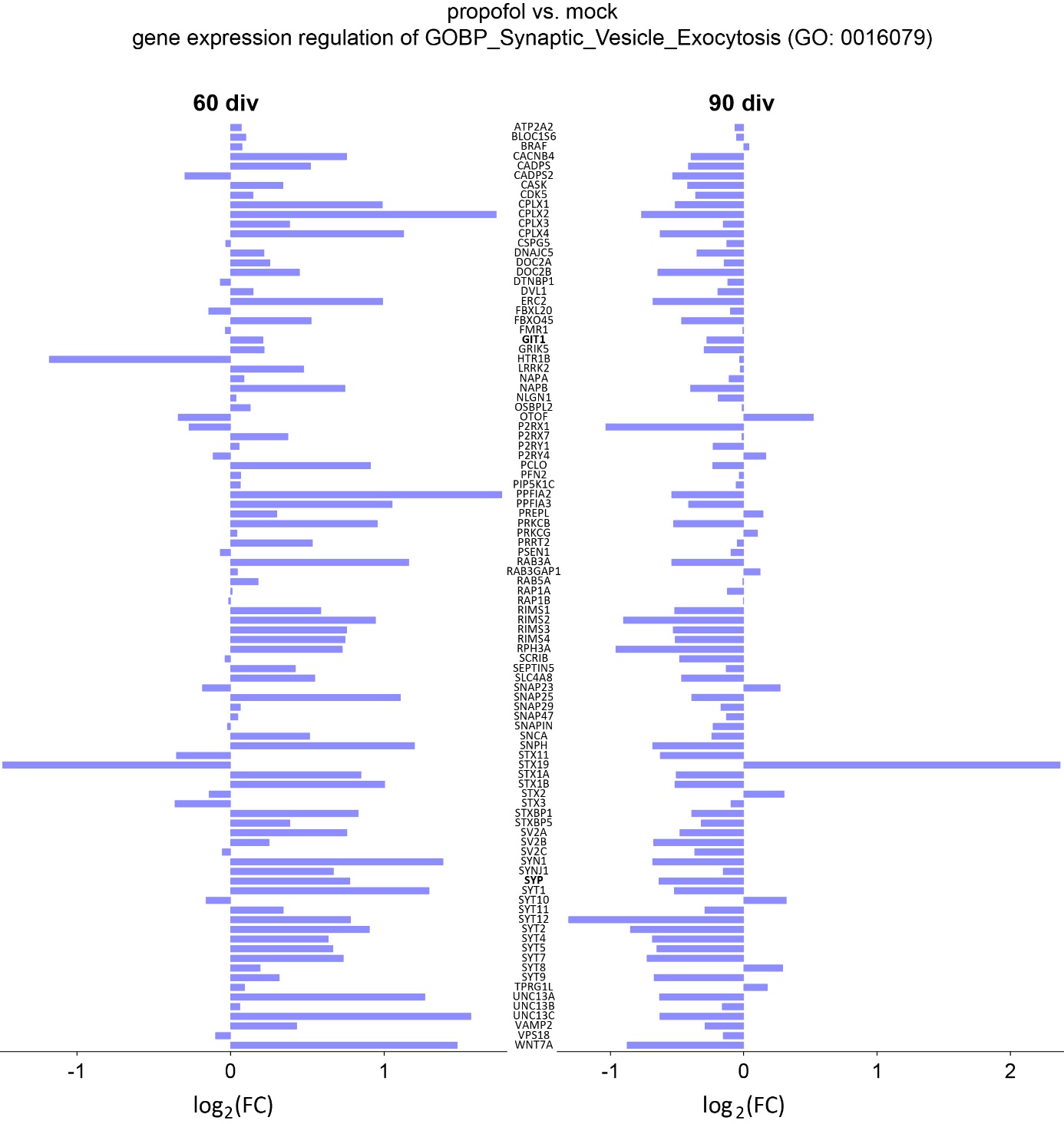


**Fig. S3: Propofol induces opposing expression of genes associated with synaptic vesicle exocytosis.** Bar plots showing the log_2_-transformed fold change of individual genes expression belonging to the GOBP_Synaptic_Vesicle_Exocytosis (GO: 0016079). Each horizontal bar represents a gene, with the direction and magnitude of expression change indicating along the X-axis. Positive values represents upregulation, while negative values represents downregulation in propofol treatment, respectively for different stages (60 or 90 div).

Bar plots showing the log_2_-transformed fold change of individual genes expression belonging to the GOBP_Synaptic_Vesicle_Exocytosis (GO: 0016079). Each horizontal bar represents a gene, with the direction and magnitude of expression change indicating along the X-axis. Positive values represents upregulation, while negative values represents downregulation in propofol treatment, respectively for different stages (60 or 90 div).

**Permalinks of the STRING-DB based analyses**

Fig. 5A-C, 90 div vs. 60 div (mock)

<https://version-12-0.string-db.org/cgi/network?networkId=basyT707J59P>

Fig. 5D, 60 div propofol vs. mock

<https://version-12-0.string-db.org/cgi/network?networkId=bcEYTWdWPRTt>

Fig. 5E, 90 div propofol vs mock

<https://version-12-0.string-db.org/cgi/network?networkId=bulDcUTlzx5v>
